# A Researcher's Journey Into Medical Miracles: Insights From 60 Cases of Radical Remission

**DOI:** 10.7759/cureus.105293

**Published:** 2026-03-16

**Authors:** Naida Alexander

**Affiliations:** 1 Integrative Medicine, Quantum University, Honolulu, USA

**Keywords:** belief and health, complementary therapies, emotional healing, integrative medicine, mind-body medicine, patient-centered care, radical remission, self-healing, spirituality and health, spontaneous healing

## Abstract

Background

Radical remission refers to the unexpected and often medically unexplained recovery from chronic or terminal illnesses, including cases where conventional treatment has failed or been declined. Historically, such remissions have been viewed with skepticism and labeled anecdotal. However, emerging evidence suggests these cases may reflect a complex interplay of psychological, emotional, behavioral, and spiritual factors. Documenting and analyzing such experiences can provide valuable insights into nonlinear healing processes that challenge conventional biomedical paradigms.

Objective

This study aimed to examine recurring psychological, emotional, behavioral, and integrative health-related factors reported by individuals who experienced radical remission. It focused on recurring themes such as mindset shifts, emotional processing, lifestyle changes, and beliefs, including the use of integrative medicine approaches alongside conventional treatment.

Methods

A retrospective, observational study was conducted involving 60 individuals who reported either full or significant remission from severe or incurable conditions, including late-stage cancers, autoimmune diseases, and multi-organ failure. Participants completed a structured, IRB-approved questionnaire that examined demographic background, disease history, conventional and complementary treatment usage, psychological states, emotional processing, and spiritual or existential experiences. Responses were summarized using descriptive analysis, and recurring psychological, behavioral, and integrative health-related factors were identified through thematic coding.

Results

Five dominant themes emerged across the dataset: (1) an unwavering belief in the possibility of healing, despite medical prognosis; (2) intentional emotional work and release of unresolved trauma; (3) reconnection with a deeper sense of life purpose or meaning; (4) active engagement with complementary and alternative therapies as part of a holistic approach; and (5) a profound spiritual or existential transformation. These findings point to healing as a multifactorial, individualized process driven as much by internal transformation as by external action.

Conclusion

The study highlights the need for a broader, integrative framework in healthcare - one that includes emotional, cognitive, and spiritual dimensions alongside physical treatment. Radical remission may not be a random phenomenon but rather the outcome of deep psychological shifts and intentional life changes. Recognizing and studying these experiences can enrich patient-centered care, inform clinical practice, and open new avenues for research into the dynamics of healing.

## Introduction

In modern biomedicine, recovery from advanced or incurable illness is typically expected to follow predictable, measurable patterns. Yet across cultures and clinical contexts, there have been recurring accounts of individuals who experience remission from terminal or chronic diseases without a clear medical explanation - a phenomenon increasingly referred to as radical remission [1,2]. These cases, though rare, challenge the limits of conventional medical understanding and prompt deeper inquiry into the biological, emotional, and psychosocial dimensions of healing.

Radical remission is defined as the full or significant reversal of a disease, often after conventional medical treatment has failed, been declined, or when non-mainstream therapies have been pursued, without a sufficient explanation based on current scientific understanding [3,4]. While spontaneous remission has historically referred to unexplained tumor regression in oncology and immunology [5], radical remission encompasses a broader phenomenon that includes rare diseases and considers emotional, spiritual, behavioral, and integrative factors as potential contributors to healing.

Despite their potential value, these cases remain largely underexplored in the medical literature, in part due to their anecdotal nature and the absence of a clear mechanistic framework. However, recent work by researchers such as Turner [3], Radin [6], and Wahbeh [7] has begun to document patterns among individuals who report radical healing, many of whom report engaging in lifestyle changes, complementary therapies, and profound shifts in personal beliefs and purpose.

A retrospective, questionnaire-based qualitative study was conducted involving 60 individuals who reported radical remission from serious medical conditions, including stage IV cancers, autoimmune disorders, neurological diseases, and multi-organ failure. Structured survey instruments and thematic analysis were used to examine participants’ reported use of conventional and complementary therapies, as well as emotional and spiritual practices, and the psychological and behavioral factors adopted during the period preceding their recovery. Although the full statistical analysis is not presented in this article, the current work provides a thematic synthesis of the principal patterns that emerged from these data.

These findings contribute to the growing body of literature suggesting that healing outcomes may be influenced by a combination of medical, psychological, behavioral, and experiential variables. By synthesizing patient-reported factors associated with radical remission, this article aims to support a broader understanding of the potential mechanisms contributing to unexpected or atypical recovery trajectories.

## Materials and methods

This study did not involve direct research with human subjects and, therefore, did not require Institutional Review Board (IRB) approval. The project was reviewed and approved by the Dissertation Committee of the International Quantum University for Integrative Medicine, which serves as the ethical oversight body for student research. The committee determined that ethics permissions were not required, as the study involved analysis of existing literature and retrospective, non-identifiable questionnaire data. The research was conducted in accordance with the University’s Academic Integrity and Conduct Policy and adhered to the principles of the Declaration of Helsinki.

Study design and participants

A retrospective, questionnaire-based study was conducted with individuals who reported experiencing radical remission from chronic or life-threatening illnesses. Participants were included if they self-reported that their treating physicians had characterized their disease as incurable, irreversible, or associated with a poor prognosis.

Recruitment occurred between July 2020 and September 2022, using personal contacts, word-of-mouth, and outreach through social media networks. A voluntary, purposive sampling approach was used. To be eligible, participants had to (1) self-report a physician-diagnosed chronic or incurable condition, (2) self-report full or significant remission, and (3) complete at least 80% of the structured survey. A total of 60 participants met the inclusion criteria.

Data collection tool

Data were collected via an online questionnaire developed for this study. Responses were analyzed through manual thematic coding: participant narratives were read repeatedly, initial codes were generated, and related codes were grouped into broader psychological, emotional, behavioral, and integrative themes. These themes were then synthesized narratively to summarize the most frequently reported healing factors. The survey included (1) demographic information; (2) disease and recovery details (diagnosis, recovery timeline); (3) treatment modalities used (conventional, complementary, and alternative therapies, including lifestyle practices such as nutrition changes, mind-body activities, or physical movement when reported, rated for perceived impact); and (4) personal life attitudes (emotional resilience, spiritual practices, belief, and purpose).

The survey was administered in both English and Hebrew via the SMART-TRIAL platform (Greenlight Guru Clinical; formerly SMART-TRIAL, Indianapolis, IN, USA) (Electronic Data Capture system), ensuring secure and anonymized data handling.

Analysis approach

Descriptive statistics were used in the original dataset to summarize trends in questionnaire responses. In the present article, participant responses were analyzed using thematic coding to identify recurring psychological, emotional, behavioral, and integrative factors associated with radical remission. These themes were then synthesized narratively to summarize shared patterns across participants.

## Results

**Figure 1:**
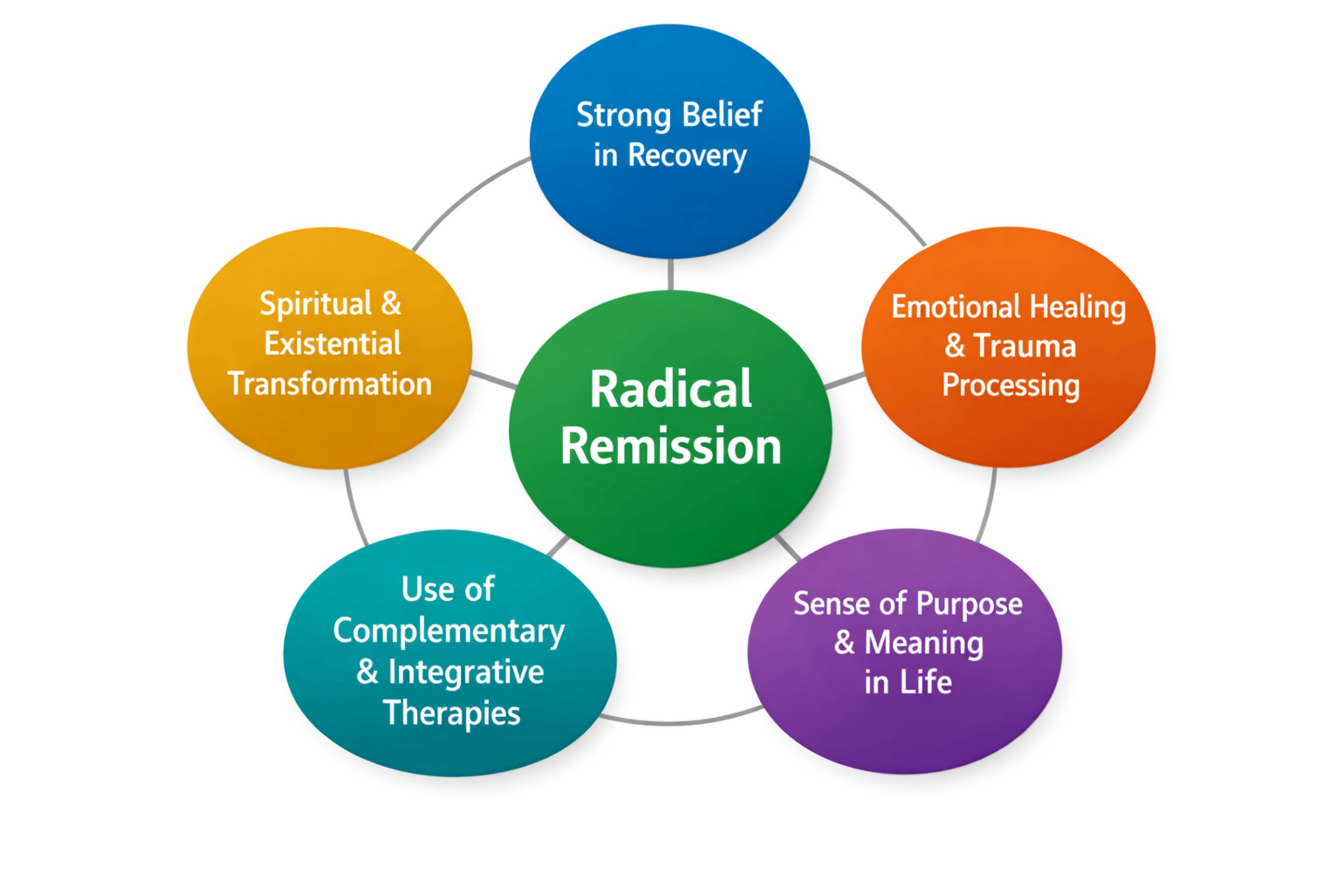
Key factors associated with radical remission identified in participant responses Image created by the authors using MS PowerPoint (Microsoft Corporation, Redmond, Washington).

Although each participant’s story was unique, several recurrent themes emerged across the 60 cases of radical remission. These themes did not point to a single miraculous cure or treatment but rather to a convergence of internal transformation, emotional release, and lifestyle change, often occurring simultaneously (Figure 1; Table 1).

**Table 1:** Prevalence and perceived contribution of therapeutic and psychosocial factors reported by participants experiencing radical remission (N = 60)

Factor Domain	Specific Factor Category	Participants Reporting Use (%)	Mean Perceived Contribution (0–10)
Therapeutic approaches	Conventional medical treatments	85.2	5.37
	Traditional Eastern medicine	54.1	6.06
	Energy-based therapies	65.6	7.30
	Mind-body interventions	68.9	7.78
	Nutritional and lifestyle modifications	77.0	7.93
	Herbal and natural remedies	29.5	5.19
Psychosocial and life-attitude factors	Spiritual or religious factors	82.0	8.46
	Emotional and social support	86.9	8.49
	Positive life attitudes	95.1	9.23

Five dominant themes

Strong Belief in Healing

Over 85% of participants reported that belief in their ability to heal played a crucial role. This belief often defied medical prognosis and was rooted in a deep intuitive conviction. Some referred to it as a “knowing” or a “decision to live.”

Emotional Healing and Release of Trauma

A majority of participants engaged in emotional work through forgiveness, grief processing, or therapeutic support. This was often described as a turning point. Letting go of emotional burdens seemed to coincide with physical improvement.

Sense of Life Purpose

Many participants described rediscovering a reason to live, whether through family, spiritual calling, or personal dreams. This inner motivation correlated with sustained lifestyle changes and a proactive approach to healing.

Use of Complementary and Alternative Therapies

Mind-body practices (e.g., meditation, yoga), nutritional changes, energy-based healing, and natural remedies were commonly used. These were typically integrated alongside conventional medicine and seen as part of a personal healing ecosystem.

Spiritual Awakening or Existential Shift

Spiritual practices, including prayer, surrender, and connection to something greater, were deeply influential. For many, healing was described as a spiritual journey that transformed their understanding of life and illness.

## Discussion

The findings of this study suggest that radical remission is not merely a random or inexplicable event, but rather a multifactorial process involving emotional resilience, psychological shifts, spiritual insight, and lifestyle transformation. While the biological mechanisms underlying healing remain only partially understood, research in psychoneuroimmunology has identified plausible pathways linking emotional and psychological states with neuroendocrine regulation, immune function, and inflammatory processes. At the same time, the lived experiences reported by participants point to consistent internal and external changes that accompanied recovery from life-threatening or chronic illness.

These observations are supported by existing literature. Turner’s analysis of over 1,000 cases of radical remission revealed similar patterns: belief in healing, emotional release, spiritual practice, and lifestyle overhaul were among the nine most frequently reported factors [3]. Similarly, Ventegodt et al. documented spontaneous remissions associated with emotional and existential healing processes [4]. These studies, along with the current findings, emphasize that healing may be catalyzed not only by external interventions but also by profound shifts in one’s inner world.

One of the most significant themes in this study was the strong belief in the possibility of healing. This aligns with studies in psychoneuroimmunology that have demonstrated that hope, positive expectations, and meaning-making can influence immune modulation and cellular function [8,9]. While belief alone is not a substitute for medical care, it may act as a powerful amplifier of treatment effects or even as a trigger for unanticipated healing responses.

The role of emotional healing, especially the resolution of past trauma and repressed emotion, is increasingly recognized in the integrative medicine field. Emotional suppression has been linked to chronic sympathetic nervous system activation, inflammation, and disease progression [10,11]. Participants in this study who experienced profound emotional release often reported subsequent improvement in physical symptoms, suggesting a meaningful mind-body connection that warrants deeper study [9,10].

Another critical theme was the presence of a life purpose or strong motivation to live. Existing research shows that individuals with a defined sense of purpose tend to have lower mortality rates, reduced inflammation, and greater resilience in the face of illness [12,13]. In this study, those who described their recovery as "mission-driven" often reported higher energy, better compliance with healing routines, and greater emotional stability throughout their process.

Spirituality, too, emerged as a potent dimension in the remission process. For many, spiritual awakening or reconnection was not peripheral but central to their healing. This reflects findings from Balboni et al., who reported that spiritual well-being strongly correlates with quality of life, coping, and end-of-life decision-making [14]. Importantly, this connection was observed across religious and non-religious participants, suggesting that the therapeutic value may lie not in dogma but in an inner shift toward meaning, connection, and surrender.

While most participants used complementary and alternative therapies, they did not view any single modality as curative. Rather, healing was described as a synergy of internal and external choices - emotional work, nutrition, energy-based therapies, and self-guided exploration. This integrative mindset is echoed in the literature supporting multi-dimensional care models for chronic disease management [1].

Several limitations should be considered when interpreting the findings of this study. The sample was self-selected, and the study relied primarily on retrospective self-reported information, which may introduce recall bias. In addition, medical diagnoses, treatments, and remission outcomes were not independently verified through medical records, limiting the ability to confirm disease history and clinical timelines. The absence of a control group also prevents causal interpretation of the reported factors.

The study may also be subject to survivorship bias, as it includes only individuals who reported experiencing radical remission. Consequently, the findings reflect the experiences of this specific group and should be interpreted cautiously when considering broader patient populations. Furthermore, alternative explanations for some reported remissions cannot be excluded, including potential misdiagnosis, variations in the natural course of disease, or undocumented conventional medical interventions.

More broadly, research on radical remission is challenged by incomplete documentation and potential publication bias, as unusual recovery cases are often reported anecdotally rather than systematically recorded in clinical settings. Future research using prospective designs, standardized documentation, and larger case registries will be important to strengthen the scientific understanding of this phenomenon.

## Conclusions

The experiences of individuals who achieved radical remission highlight healing as a multidimensional process involving belief, emotional transformation, spiritual growth, and integrative practices. While conventional treatments remain crucial, these cases suggest that internal shifts, such as purpose, faith, and emotional resilience, may play a vital role in recovery. From a clinical perspective, these findings suggest that healthcare professionals may benefit from exploring patients’ psychosocial and spiritual resources as part of comprehensive care. In practice, this could include encouraging supportive relationships, discussing sources of meaning or purpose, and integrating evidence-based mind-body practices such as meditation, stress-reduction techniques, or lifestyle interventions alongside standard medical treatment. Such approaches may help address the emotional and existential dimensions of serious illness while maintaining a strong foundation in evidence-based care.
